# The Effects of Fiber Inclusion on the Evolution of Desiccation Cracking in Soil-Cement

**DOI:** 10.3390/ma14174974

**Published:** 2021-08-31

**Authors:** Yaxing Xu, Xin Yao, Yan Zhuang, Wei Duan, Xidong Zhang, Shunlei Hu, Xiaoqiang Dong

**Affiliations:** 1 College of Civil Engineering, Taiyuan University of Technology, Taiyuan 030024, China; Star_Xu@tom.com (Y.X.); yaoxintyut@163.com (X.Y.); duanwei@tyut.edu.cn (W.D.); zhangxd123456@163.com (X.Z.); hushunlei97@163.com (S.H.); 2 College of Civil Engineering, Southeast University, Nanjing 211189, China; joanna_zhuang@163.com

**Keywords:** fiber inclusion, soil-cement, desiccation cracking, digital image correlation

## Abstract

Desiccation cracking frequently occurs in mud, clay, and pavement. Understanding the evolution of desiccation cracking may facilitate the development of techniques to mitigate cracking and even prevent it from developing altogether. In this study, experimental investigations were performed focusing on the effects of fibers on the evolution of desiccation cracking in soil-cement. Varied types of fibers (i.e., jute fiber and polyvinyl alcohol fiber (PVA)) and fiber contents (i.e., 0%, 0.25%, 0.5%, and 1%) were involved. The digital image correlation (DIC) method was employed to capture the evolution and propagation of cracks in the soil-cement specimens when subjected to desiccation. The results show that the presence of fibers imposes significant effects on the crack propagation pattern as well as the area and length of the cracks in the soil-cement during shrinkage. The addition of fibers, however, insignificantly affects the evaporation rate of the specimens. The crack area and crack length of the specimens decreased significantly when more fibers were included. There were no macroscopic cracks observed in the specimens where the fiber content was 1%. The DIC method effectively helped to determine the evolution of displacement and strain field on the specimens’ surface during the drying process. The DIC method is therefore useful for crack monitoring.

## 1. Introduction

The technique of soil-cement is widely used in engineering works (e.g., foundation treatment [1,2], roadbed improvement [3,4,5], soft soil reinforcement [6,7,8,9], and seepage prevention [10,11]) due to its advantages of low cost, practicality, convenient construction, and low permeability. However, soil-cement tends to shrink during dewatering and expand during watering, which may cause cracks to form. Exploring the mechanism of the evolution of desiccation cracks and then build potential techniques to reduce cracks is practical and meaningful.

Many studies have been conducted on the drying shrinkage of soils, focusing on different influencing factors such as layer thickness [12,13,14,15], size [16], moisture content [17,18,19], evaporation rate [20,21], roughness of the bottom contact surface [22], and temperature [23]. Zeng et al. [12] showed that the influence of interface friction and layer thickness has an obvious coupling effect on the drying and cracking of soil. Khatun et al. [15] showed that the cumulative total area of cracks increases along with the increase of layer thickness. Uday et al. [21] showed that the evaporation rate of soil was not affected by the initial water content and boundary conditions, but it was related to its thickness and temperature.

To reduce the adverse effects of desiccation cracking on the engineering properties of fine-grained soils and improve soil strength and resistance, researchers have used field and experimental investigations to study the effects of adding fibers (i.e., polyester fiber [24], flax fiber [25], straw fiber [26], nylon fiber [27]), sugarcane pith [14], and microorganisms [28]) and other materials to strengthen the soil. The results indicate that the average length, width, spacing, and total area of soil cracks decrease obviously with the addition of fibers. The crack intensity factor, which is defined as the ratio of the area of cracks over the total surface area, decreases with the increase of fiber content. Once the fibers are included, a network of fine cracks develops on the surface of the specimens, and the integrity of the specimens somehow improves. Wang et al. [26] used the digital image correlation (DIC) method to continuously measure the total strain on the surface of soil specimens and showed that the stress distribution around cracks could be reliably observed through strain field analysis. They also showed that most of the crossing cracks intersect orthogonally. In addition, the DIC method showed that adding fibers into clay could significantly reduce the main strain and cracking of the clay. Therefore, fiber is considered to be a useful material to reinforce soil. Here, the strengthening effect of natural jute fiber and synthetic PVA fiber on soil-cement is investigated.

Published works mainly focused on the mechanisms of dry shrinkage, the cracking of clays, and expansive soils, targeting different factors, i.e., water evaporation, strain field distribution, and crack characteristics. However, very few studies have been performed on the shrinkage and cracking of silts, which have the negative characteristics of forming mud in the presence of water and forming ash in its absence. Reinforcing soil-cement using discrete fibers has also been seldomly investigated in the literature.

In this study, the DIC method was adopted to evaluate the effects of the addition of fibers on mitigating desiccation cracking in cemented soil. Varied types of fibers and fiber contents were involved. The mechanism of desiccation cracking in fiber-reinforced soil-cement was explored.

## 2. Materials and Methods

### 2.1. Materials

**Figure 1:**
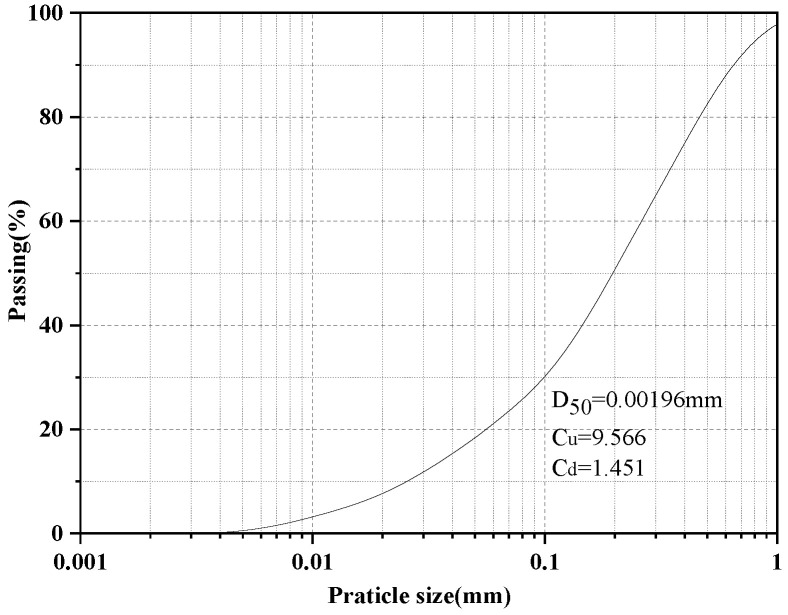
The particle size distribution of soil.

The tested soil was collected from a construction site in Taiyuan (Shanxi province of China). The index properties are listed in Table 1 and the particle size distribution of soil in Figure 1.

**Figure 2:**
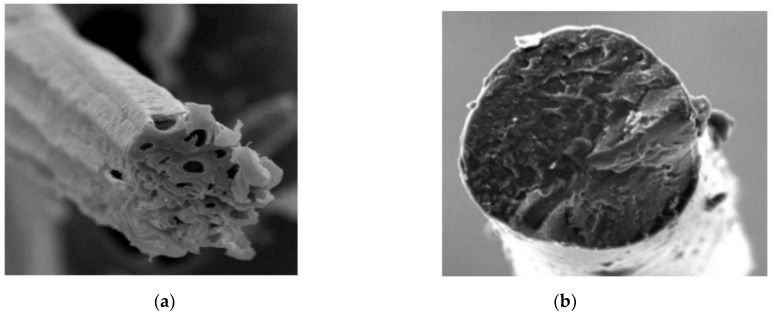
SEM images of jute and PVA fiber: (**a**) jute fiber (50 μm); (**b**) PVA fiber (100 μm).

Two types of fibers were used to reinforce soil in this study. Figure 2a,b show the SEM images of a jute fiber and PVA fiber, respectively. The jute fiber is in the shape of a slender tube. The cavity inside the jute fiber is round or oval, while the cross-section is mostly pentagonal or hexagonal. The cross-section of the PVA fiber is round and the surface is relatively smooth. The properties of the fibers are given in Table 2. Ordinary Portland cement (42.5R) was used for the cements in this study.

### 2.2. Specimen Preparation

The soil was oven-dried, crushed, and sieved through a sieve with an opening of 2 mm. The ratio of the cements added was 15%, which is the percentage of the dry weight of cements over the dry weight of the soil. Varied fiber contents (0%, 0.25%, 0.5%, and 1% by dry weight of the soil) were used to reinforce specimens, while the fiber content represents the ratio of weight of the fibers over the dry weight of soil. The components included in each mixture are given in Table 3.

The cements and soil were fully mixed in dry conditions in a blender, and then fibers were added into the mixture gradually by hand. The fibers were blown using an air gun to avoid aggregation. Thereafter, enough water was added to produce a soil-cement slurry, which was then poured into a borosilicate glass mold with a diameter of 95 mm. Immediately after that, the specimens were vibrated for 1 min to expel bubbles. The height of the specimens was 8 mm, i.e., an aspect ratio of 11.88 was maintained. Colina et al. [29] suggested that the minimum aspect ratio for cracks is about 5.8.

### 2.3. Experimental Method

**Figure 3:**
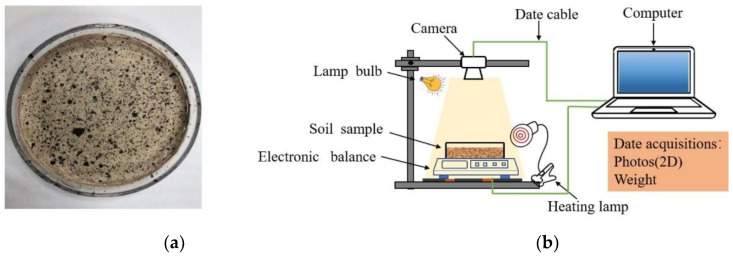
Schematic representation of the drying shrinkage crack test setup: (**a**) speckle specimens; (**b**) schematic diagram of test device.

The evaporation of water from a fresh soil-cement matrix may lead to deformation of the speckles on the specimen. In this study, speckles were sprayed on the freshly prepared slurry after drying at room temperature for half an hour. Figure 3a shows the speckle pattern on the surface of the specimens. The DIC images were obtained using a charge-coupled device (CCD) camera with a resolution of 10 million pixels. A heating lamp was used to heat the specimens. The temperature of the specimens’ surface was controlled at 50 °C through an infrared temperature gun. The relative humidity was maintained at 20% RH. LED light sources were placed on both sides of the device. To investigate the evaporation rate of water during the desiccation process, an electronic balance (with an accuracy of 0.1 g) was used to weigh the specimens before and after evaporation. The variation in the moisture content of the specimens was then calculated according to the obtained mass loss.

For the DIC analysis, post-processing of the images was conducted using GOM Correlate 2019 software to quantitatively analyze the effects of the addition of fibers on the early strain field of the soil-cement samples, as well as the area and length of the desiccation cracks. Due to the continuous evaporation of water in the specimens, the contrast of the captured images was reduced. In advance of the post-processing, the brightness of all images was reduced and the contrast was increased using Photoshop software. The images were taken once every minute over a time span of 300 min. The size of subsets and step sizes were adjusted according to the results of the pattern quality in the DIC software. The size of subsets here is 50 × 50 pixels, and the center distance between subsets is 7 pixels.

## 3. Results and Discussion

### 3.1. Water Evaporation during the Drying Process

**Figure 4:**
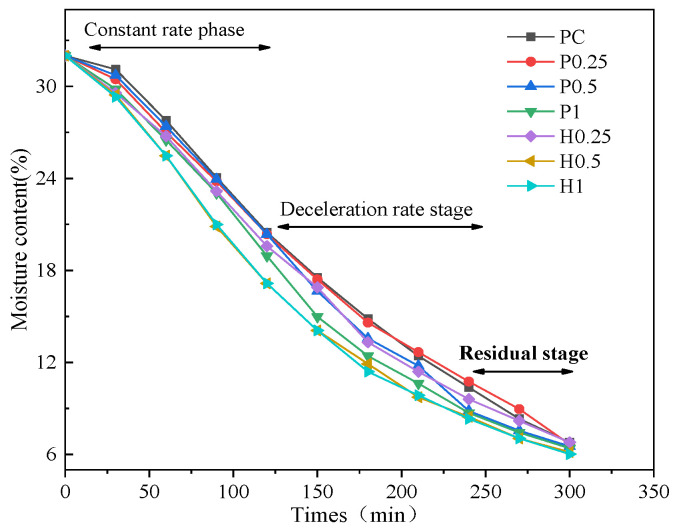
Decrease of the moisture content of the specimens with time elapsing.

There is a close coupling relationship between the propagation of cracks and the loss of water. The evaporation rate affects the propagation rate of cracks and the crack pattern. At the same time, the propagation rate of cracks accelerates the evaporation rate. Figure 4 shows the variation of the moisture content in the specimens during the drying process.

It can be observed that the evaporation process in the specimens of each group shows the same trend. The evaporation rate is not affected by the fiber type and the fiber content. The addition of fiber has an insignificant effect on the drying and water loss process. The shrinkage process during drying of the specimens may be divided into three stages: the constant rate stage, the deceleration rate stage, and the residual stage.

In the initial stage of drying, the specimen is in the constant rate stage. The moisture content decreases linearly with the increase in the desiccation time. The water that is supplied to the soil–water interface for evaporation also decreases gradually. Moreover, with the increase in the matric suction, the resistance to the movement of the water molecules becomes greater. Therefore, the evaporation rate decreases gradually, and the curve shows a turning point after which the slope decreases. It can be seen in Figure 3 that the moisture content of the PC specimen in the residual stage is slightly lower than P0.25, which may be caused by the larger crack area. The water retention of jute fiber is lower than that of PVA fiber, which may be because the jute fiber is a natural fiber. The SEM image of the jute fiber shows a porous structure consisting of multiple lumens [30,31]. As a consequence, jute fiber has good moisture absorption performance and disperses water quickly.

### 3.2. Evolution of Cracking in the Plain Cement Soil

**Figure 5:**
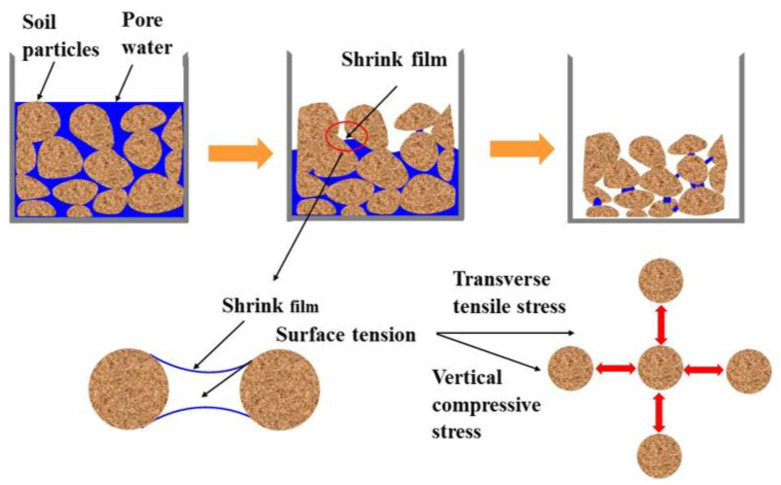
Schematic description of mechanism leading to formation of desiccation cracking of plain soil-cement [32].

Figure 5 is a schematic diagram showing the dry cracking of the plain soil-cement [32]. The initiation of cracks is caused by the evaporation of moisture from the surface of the specimens. In the initial stage of drying, the moisture content is large, the soil-cement particles are completely immersed by free water, and the specimens are in the form of fluid mud. At this stage, the deformation in the specimens is mainly vertical settlement. With the elapsing of drying time, as the free water between the particles begins to evaporate, the capillary water effect may be triggered. Water in the lower section of the specimens continuously migrates to the upper water–air interface. The moisture content of the specimens decreases continuously. The formation of capillary water bridges and the increase in the pore water surface tension leads to the formation of a meniscus (also denoted to be shrink film) between the surface pore water and the soil-cement particles. The surface tension of the shrink film exerts a certain horizontal tensile stress and vertical compressive stress on the soil-cement particles, which is coupled with the capillary water pressure and gravity, causing the soil-cement particles to move close together in the horizontal direction, with consolidation occurring in the vertical direction. Each soil-cement particle on the surface is subjected to the tension induced by the capillary water of the surrounding particles. A tensile stress field is formed in the upper layer. Once the tensile stress generated in the soil-cement exceeds the tensile resistance of the soil-cement, desiccation cracks develop on the surface and the tensile stress is released. Therefore, it can be inferred that matrix suction and tensile strength are the two main factors controlling the dry cracking of soil-cement.

**Figure 6:**
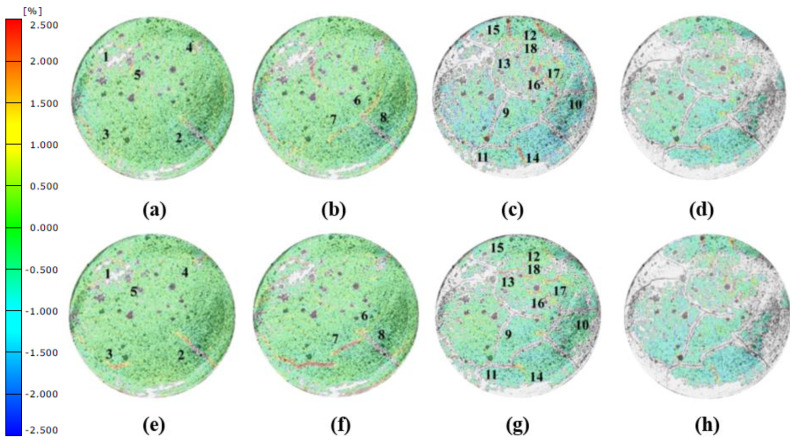
Strain of plain cemented soil in the early times: (**a**) ε_x_-50 min; (**b**) ε_x_-60 min; (**c**) ε_x_-80 min; (**d**) ε_x_-100 min; (**e**) ε_y_-50 min; (**f**) ε_y_-60 min; (**g**) ε_y_-80 min; (**h**) ε_y_-100 min.

Nahlawi et al. [14] reported that drying shrinkage cracks can be categorized into main cracks, secondary cracks, and tertiary cracks based on the progressive order of their occurrence. It can be seen from Figure 6 that with the evaporation of water, when the cracks generated from the edge expand and widen to the center, secondary cracks are generated and expand. The direction of propagation of the secondary cracks seems to remain perpendicular to the main crack and parallel to the edge. As more time elapses, the cracks’ length mostly becomes stable, while the cracks’ width continuously increases. The development of the cracks approaches stability after 100 min. The crack network mainly includes three types of polygons, i.e., triangles, quadrilaterals, and pentagons. The crack segments intersect with each other, and the intersection angles approximately range from 90° to 150°. This phenomenon may be explained by the maximum stress release criterion. When a crack develops, the surrounding tensile stress is gradually released. The direction of the maximum tensile stress is parallel to the direction of the crack. The crack tends to expand along the direction perpendicular to the local maximum main stress. Therefore, the secondary cracks are perpendicular to the primary crack and intersect with the tertiary crack.

### 3.3. Deformation Field of the Fiber-Reinforced Soil-Cement during Drying Shrinkage

The post-processing software used in this study provides information about the surface deformation of fiber-reinforced soil-cement specimens during drying. The strain data is the value of the partial derivative of the displacement, so the displacement gap is represented as the local extreme value of the strain, i.e., the surface crack.

**Figure 7:**
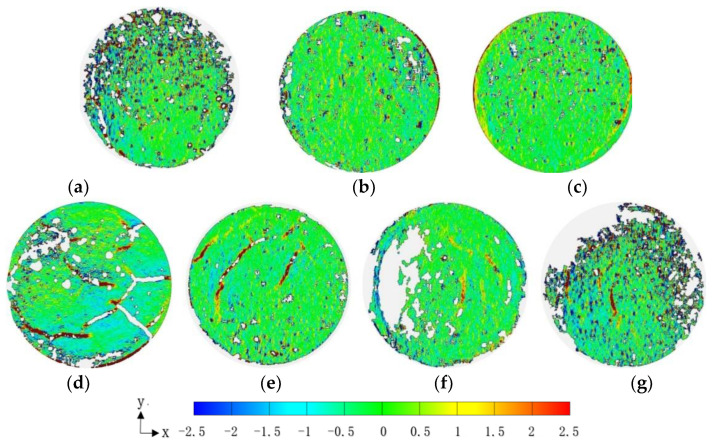
Strain field along the X-direction of the specimens at 70 min: (**a**) ε_x_-PC; (**b**) ε_x_-H0.25; (**c**) ε_x_-P0.25; (**d**) ε_x_-H0.5; (**e**) ε_x_-P0.5; (**f**) ε_x_-H1; (**g**) ε_x_-P1.

The X-direction strain field distributions of the jute fiber and PVA fiber-reinforced soil-cement specimens were measured at 70 min, and the results are shown in Figure 6. The cracks observed in Figure 7a are mainly confined to the high tensile region. The edges of the cracks have a high tensile strain (the strain field is shown in red), and the strain field at the corner of two intersecting cracks is even higher (dark red). The occurrence of desiccation cracks leads to a redistribution of the stress, and the strain field in the area adjacent to the cracks is negative, resulting in a greater compressive strain. As can be seen from Figure 6b,d, three disjoint cracks developed in the specimen reinforced with 0.25% jute fiber, indicating that the addition of fiber changes the propagation direction and stress distribution of cracks. Disjoint cracks also appeared in the middle of the specimen reinforced with 0.5% jute fiber, but the crack length and strain were significantly smaller than those in the H0.25 specimen. Similar to the jute fiber specimens, three cracks developed in the middle of the P0.25 specimen. The strain at these cracks was between 1% and 2.5%, while the lengths of these cracks were shorter than that in the H0.25 specimen. In addition to the cracks that developed at the edges of the P0.5 specimen, short disjoint non-perforating macro-cracks developed in a scattered manner in the middle of the specimen, and the P0.5 specimen had relatively dispersed and uniform micro-cracks. The strain distribution in the H1 and P1 specimens was uniform, and no macro-cracks were found in the middle of these specimens, except at the edge of the container. It can be seen that the addition of fibers altered the propagation direction and stress distribution of cracks. The development of cracks in the specimens with fiber initiated parallelly from the middle of the specimens. The increase of the fiber content was able to reduce the length and width of the cracks.

**Figure 8:**
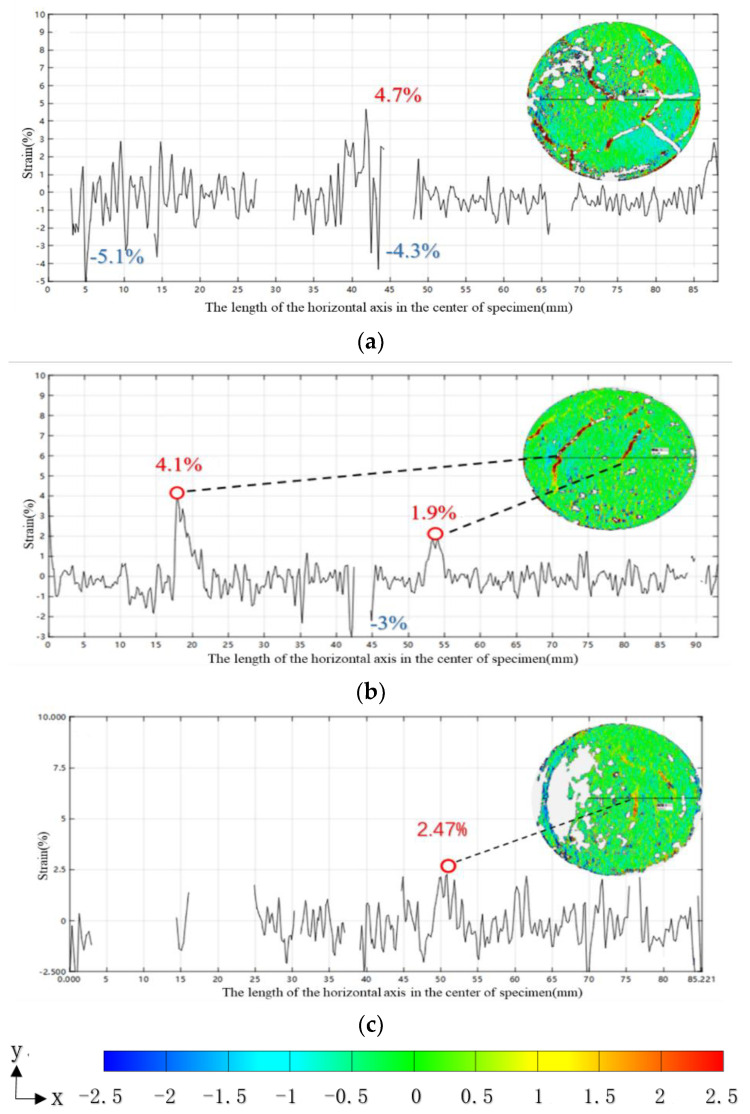
The strain curve along the X-direction of the horizontal axis of the specimen center at 70 min: (**a**) ε_x_-PC; (**b**) ε_x_-H0.25; (**c**) ε_x_-P0.25.

A horizontal line through the center point of the specimens may be drawn to represent the positions of interest, as shown in Figure 8. The strain values in the X-direction of the horizontal axis of the specimen’s center, changing with their position along the drawn horizontal line, are shown in Figure 7. It can be seen from Figure 7 that the maximum tensile strain at the center of the crack in the plain soil-cement is 4.7%. The tensile strain caused by the crack position on the horizontal axis is between 0~4.7%, while the range of compressive strain is between 0~5.1%. For the H0.25 specimen, the maximum tensile strain at the crack at the center line of 18 mm is 4.1%, and the tensile strain at the crack at the center line of 54 mm is 1.9%. Except for the crack areas, the tensile strain at the crack position is in the range of 0~1.3%, and the average tensile strain is reduced by the inclusion of fiber. The large compressive strain on the center line is located at 36 mm, 43 mm, and 45 mm, which is different from that of plain soil-cement where the larger compressive strain is not located around the crack. The maximum tensile strain of the specimens is 2.47% at P 0.25. Figure 7b,c show that the addition of the two types of fibers to the soil-cement reduces the tensile and compressive strains in the peak and non-peak areas of the soil-cement cracks and that the fiber reduces the stress concentration around the cracks by transferring the stress to the soil-cement matrix. In addition, it can be noted from the figure that the location of the strain peak is consistent with the location of the cracks.

### 3.4. Characterization of Cracks in the Fiber-Reinforced Soil-Cement

**Figure 9:**
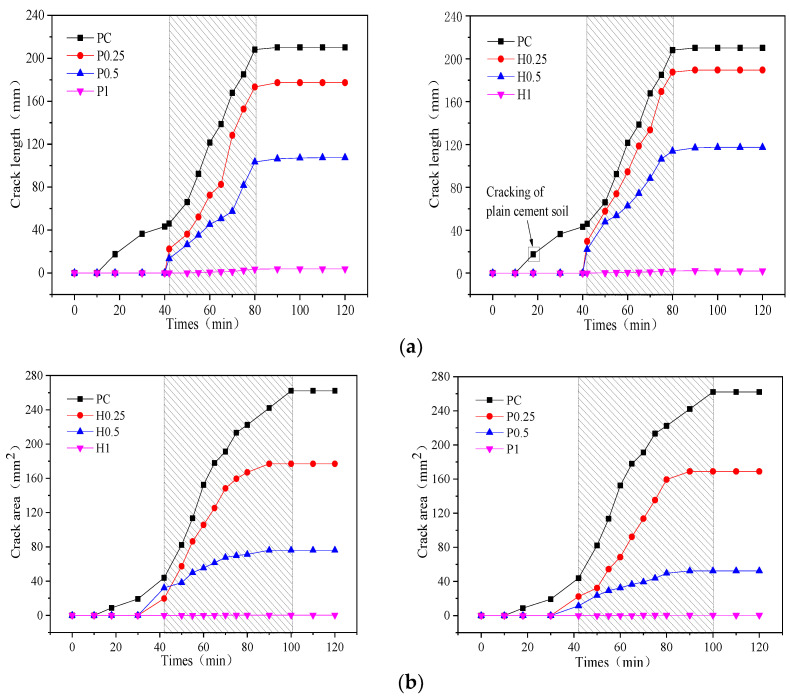
Quantitative results of desiccation crack: (**a**) curve of the crack length versus time; (**b**) curve of the crack area versus time.

The crack length and crack area were investigated to get to know the mechanism of desiccation cracking. The crack length is regarded as the total surface crack length, and the crack area is regarded as the total surface crack area. Figure 9a,b show the curves of the total crack length and crack area of the specimens during the drying process. The addition of the two types of fibers reduces the crack length and crack area. Cracks appear in the plain cement soil specimens 18 min after the initiation of the drying. The time spent on the formation of the first crack in the specimens reinforced with two fibers when fiber content is 0.25% is delayed. The crack length in the specimens including jute fibers of 0.25% and 0.5% decreases by 11.1% and 32.5%, respectively. The crack length in the specimens with 0.25% and 0.5% PVA fiber decreases by 16.8% and 47.7%, respectively, when compared with that in the plain soil-cement. When compared with the plain soil-cement, no macroscopic cracks were observed in the specimens with 1% jute and PVA fiber. It can be inferred that the addition of fiber can significantly reduce desiccation cracking in soil-cement.

**Figure 10:**
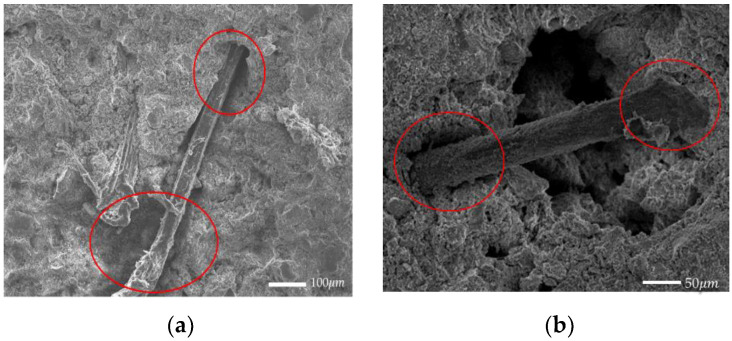
SEM images of fiber and soil-cement matrix interface: (**a**) jute fiber; (**b**) PVA fiber.

The inclusion of fibers reduces desiccation cracking in soil-cement, because fibers can reduce the tensile stress generated in the cement material by interacting with the matrix, and they can also transfer the tensile stress from the matrix to themselves; and it can be seen from Figure 10 that there is a considerable gap between jute fiber and the surrounding matrix, indicating poor fiber–matrix interfacial bonding. The interfacial gap between PVA fiber and the matrix is reduced, and the bond is close, indicating that PVA fiber and the matrix have good interfacial bond performance. The Oushabi and Chhetri [33,34] studies showed that good interfacial bonding ensures load transfer from the matrix to the fiber, which helps to reduce stress concentrations and postpones crack initiation and propagation. At the same time, PVA fiber has a larger specific surface area than jute fiber, and the formation of a strong chemical bond between the hydroxyl group in its molecular structure and the matrix of the soil-cement makes it slightly better than jute fiber in reducing desiccation cracks.

## 4. Mechanism of Desiccation Cracking of Fiber-Reinforced Soil-Cement

**Figure 11:**
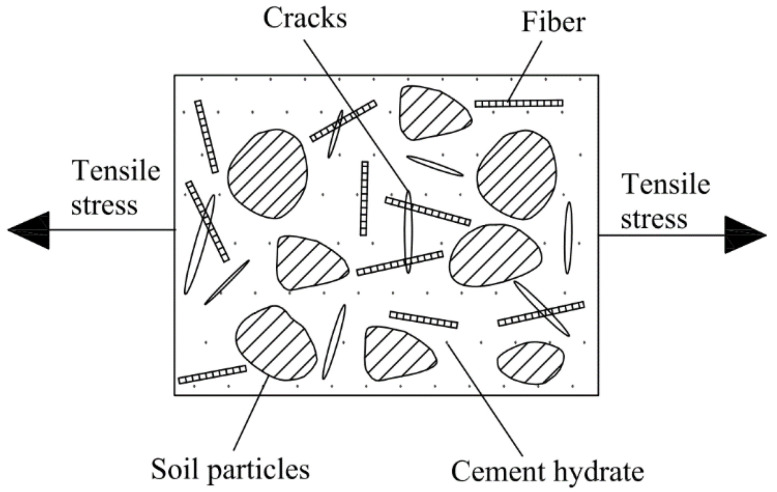
Model of fibers restraining crack formation in soil-cement.

**Figure 12:**
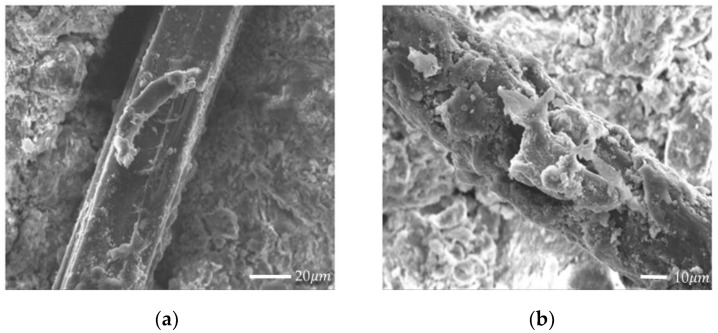
Interface between the fiber and soil-cement: (**a**) jute fiber (×800); (**b**) PVA fiber (×1000).

It should be noted that the desiccation cracking of soil-cement is a form of tensile failure. When the tensile stress caused by the suction of the matrix exceeds the tensile strength of the soil-cement, desiccation cracking occurs. Matrix suction and tensile strength are two main factors controlling desiccation cracking. When different types and contents of fibers were added, the desiccation cracks of the soil-cement matrix significantly reduced mainly due to the following reasons:

(1) The tensile strength of fiber is much greater than that of soil-cement. After the formation of the initial cracks, the fibers in the crack area bear the tensile stress, which alleviates the stress concentration near the cracks. The interaction between the soil-cement matrix and the fibers is effective in suppressing desiccation cracking.
(2) According to Figure 11, when tensile cracks appear in the soil-cement matrix, the fibers improve the strain field of the soil-cement matrix by providing a bridge relay across the cracks, which decreases the development of the width, area, and propagation of the cracks so that more fine cracks appear rather than fewer but wider cracks. Tang et al. [35] conducted a pull-out test on a single fiber embedded in a soil matrix and found that the interfacial shear strength increased with the decrease in the water content; the increase in the interfacial shear strength helped the fiber to restrain the movement of the separated soil clumps when cracks were initiated, and the bridging effect caused by the addition of the fiber increased.
(3) Figure 12 shows that a large number of cement hydration products adhered to the surface of the fibers, which have greater strength and cementation, effectively limiting the relative motion of the fiber. Therefore, the fibers distribute the stress in a wide area and inhibit cracks from forming or propagating. Therefore, the combination of fiber and cement inclusion improves the efficiency of load transfer from the soil matrix to the fiber.

In addition, the hydration of cement binds the soil particles together and makes the soil particle structure more compact, resulting in an increase in the normal stress and effective contact area around the fiber body, thus increasing the coefficient of static friction between the fibers and the soil-cement matrix and increasing the mechanical pulling force between the fibers and the soil matrix.

## 5. Conclusions

In this study, tests on the drying shrinkage of fiber reinforced soil-cement were performed. Varied amounts of fibers and different types of fibers were engaged. The effects of fiber-reinforcement on the evolution of desiccation cracking in a soil-cement matrix were investigated by adopting the DIC method. Several conclusions may be drawn from the test results.

(1) The drying shrinkage process of fiber-reinforced soil-cement may be categorized into three stages: the constant rate stage, the deceleration rate stage, and the residual stage. The evaporation water loss curves of each group of specimens show similar trends. The moisture content is not affected by the types and contents of fibers. The inclusion of fiber does not significantly affect water loss during the drying process.

(2) The addition of fibers alters the propagation direction and stress distribution of cracks. The desiccation cracks of plain soil-cement can be categorized into main cracks, secondary cracks, and tertiary cracks. The intersection angles of the cracks in the crack network ranges from about 90° to 150°.

(3) The presence of fibers reduces the tensile and compressive strains in the peak and non-peak areas of the soil-cement cracks, and the stress concentration neighboring the cracks is improved due to stress transfer between the fiber and the soil-cement matrix. The DIC method shows that the strain peak position is consistent with the crack location.

(4) The presence of fibers delays the time spent before the formation of the first cracking. The increase of fiber content significantly reduces the length and area of desiccation cracks. There are no macroscopic cracks observed in specimens with 1% jute and PVA fibers.

## Figures and Tables

**Table 1:** Index properties of the tested soil.

Specific Gravity	Moisture Content (%)	Dry Density (g/cm^3^)	Void Ratio	Liquid Limit (%)	Plastic Limit (%)	Plasticity INDEX
2.7	18.3	1.3	1.074	25.5	17.2	9.8

**Table 2:** Properties of fibers.

Fiber	Specific Gravity (g/cm^3^)	Length (mm)	Diameter (μm)	Tensile Strength (MPa)
Jute fiber	1.45	12	100	480
PVA fiber	1.3	12	40	1600

**Table 3:** Mixture proportions.

Specimen Legend	Soil Weight (g)	Cement Weight (g)	Water Content (%)	Fiber Content (%)	Fiber Type
PC	100	15	33	0	N/A
P0.25	100	15	33	0.25	PVA
P0.5	100	15	33	0.5	PVA
P1	100	15	33	1	PVA
H0.25	100	15	33	0.25	Jute
H0.5	100	15	33	0.5	Jute
H1	100	15	33	1	Jute

## Data Availability

The data used to support the findings of this study are available from the corresponding author upon request.
